# *Toxoplasma gondii* induced cognitive impairment in rats via dysregulation of dopamine receptors and indoleamine 2,3 dioxygenase

**DOI:** 10.1016/j.heliyon.2023.e14370

**Published:** 2023-03-09

**Authors:** Mohammed Nasiru Wana, Malaika Watanabe, Samaila Musa Chiroma, Ngah Zasmy Unyah, Sharif Alhassan Abdullahi, Shariza Nordin, Rusliza Basir, Mohamad Aris Mohd Moklas, Roslaini Abd Majid

**Affiliations:** a Department of Medical Microbiology and Parasitology, Faculty of Medicine and Health Sciences, Universiti Putra Malaysia, 43400, Serdang, Selangor, Malaysia; b Department of Veterinary Clinical Studies, Faculty of Veterinary Medicine, Universiti Putra Malaysia, 43400, Serdang, Selangor, Malaysia; c Department of Human Anatomy, Faculty of Basic Medical Sciences, University of Maiduguri, Nigeria; d Department of Biomedical Sciences, Faculty of Medicine and Health Sciences, Universiti Putra Malaysia, 43400, Serdang, Selangor, Malaysia; e Department of Human Anatomy, Faculty of Veterinary Medicine, Universiti Putra Malaysia, 43400, Serdang, Selangor, Malaysia; f Department of Pre-Clinical, Faculty of Medicine and Defence Health, National Defence University of Malaysia, Kem Sungai Besi, 57000, Kuala Lumpur, Malaysia; g Department of Biological Sciences, Faculty of Science, Abubakar Tafawa Balewa University Bauchi, Nigeria; h Department of Medical Microbiology and Parasitology, Faculty of Clinical Sciences, Bayero University, Kano, Nigeria; i Newcastle University Medicine Malaysia (NuMed) No 1, Jalan Sarjana 1,Kota Ilmu, EduCity@Iskandar,79200 Iskandar Puteri (formerly Nusajaya) Johor-Malaysia

**Keywords:** *Toxoplasma gondii*, Anxiety, Fatal attraction, Gene expression, Neurotransmitters, Malaysia, Rat, Cognition

## Abstract

*Toxoplasma gondii* (*T. gondii*) is a parasite capable of residing in the brain of their host which influences behaviour changes due to alterations in the neurotransmitters. Consequently, dopamine receptors (DRD) and indoleamine 2, 3 dioxygenase (IDO) dysregulation facilitate the progression of behaviour changes in a host as a response to infection. This study tested the effect of neurotransmitter changes as a result of *T. gondii* infection on rats cognitive impairment. The *T. gondii* strain of type I, II and III from Malaysia were previously identified by standard procedures. Sporulated oocysts each of type I, II and III were inoculated separately into three groups of Wistar rats (n = 9) respectively. Two separate control groups received either phosphate buffered saline (PBS) or MK-801 (dizocilpine). Behaviour changes were evaluated at nine weeks post infection in a square box, elevated plus maze and gene expression level of DRD and IDO compounds. The study revealed increased fatal feline attraction, reduced anxiety, decreased DRD and increased IDO gene expression in the *T. gondii* infected groups and MK-801 compared to the PBS control group. In conclusion, *T. gondii* infection alter the level of neurotransmitters in rat which cause cognitive impairment. This implies that all the *T. gondii* strain can cause behaviour changes if human were infected.

## Graphical abstract

A schematic diagram of dopamine and kynurenic acid pathways in the brain of *T. gondii* infected rat.*IDO: indoleamine-2,3-dioxygenase, DRD1: dopamine receptors, L-DOPA: L-dihydroxyphenylalanine,DA: dopamine, KYNA: kynurenic acid, Kyn: kynurenine.

**Figure:**
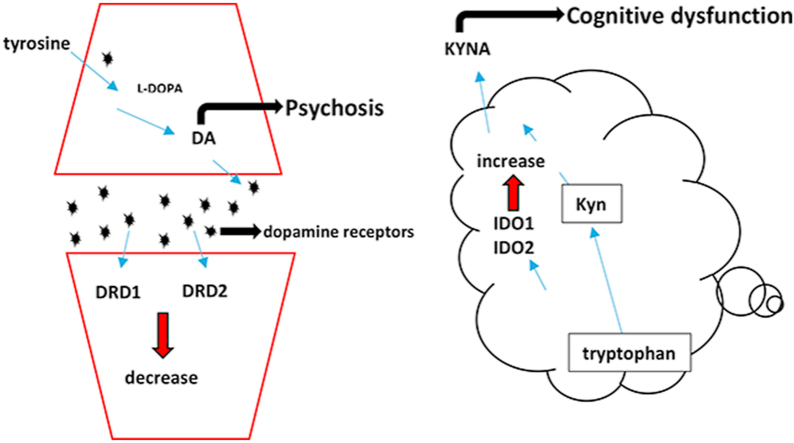

## 1 Introduction

*Toxoplasma gondii (T. gondii)* is a protozoan parasite that has been implicated as a cause of brain dysregulation of metabolic processes that can affect host behaviour [[1], [2], [3]]. There are three distinct stages of the parasite: the sporozoite infective stage, an immune-modulation tachyzoite and tissue stage bradyzoite [4]. Sporozoite found in oocyst initiates the infection which transforms quickly into the rapidly dividing tachyzoites in the blood, the acute stage followed by the chronic stage when *T. gondii* formed tissue cysts mainly in brain tissues as bradyzoites [5,6]. The tachyzoite stage, elicited the host immune response which may alter the levels of neurotransmitters in the brain [7,8] indirectly. Further, *T. gondii* bradyzoites established itself as a tissue cysts in the brain and can persist indefinitely throughout the entire life of the host [9]. However, the mechanism underlying the indirect effect of *T. gondii* tissue cysts has been proposed to involved dysregulation of neurotransmitters such as dopamine and kynurenic acid in the brain [10,11].

Dopamine is another neurotransmitter that is produced from tyrosine and transported to the dopaminergic neurons of the substantia nigra (SN) and ventral tegmental area (VTA) that mediates several aspects of behaviour deficits [12]. Further, the enzyme tyrosine hydrolase catalyse the conversion of tyrosine to dihydroxyphenylalanine (DOPA), then finally converted to DA through DOPA decarboxylase and stored in synaptic vesicles [13]. These synaptic vesicles when stimulated initiate the release of DA into the surface of pre-synapse waiting to either bind with dopamine receptors (DRD) or reuptake back to the synapse by dopamine transporters (DAT) that formed postsynapse [13]. Thus, binding between DRD and DA in the VTA produced DA signals that are transmitted to either mesocortical or mesolimbic pathways in the brain. However, DRD gene dysregulation produced hyperactivity of DA transmission in the mesolimbic area and hypoactivity of DA in the mesocortical area which are core features relevant to cause mental disorder [14]. Moreover, study have shown that *T. gondii* tissue cyst is linked to the decrease in dopamine receptors (DRD) that increase dopamine (DA) production [7,13,15].

Effect of host immune responses have been associated with behaviour changes through induction of a key enzyme, indoleamine-2,3 dioxygenase (IDO) production and tryptophan degradation that also leads to serotonin decrease in the brain [10,16]. Thus, a series of IDO dysfunction that took place mediates and altered the release of kynurenic acid from tryptophan into the brain and other body systems [16]. Further, in addition to activation of IDO, kynurenine (Kyn) is produced as a neuroactive compound which ultimately crosses the blood brain barrier and had been found as part of neurodegenerative and behaviour deficits in the brain [[16], [17], [18]].

To date, studies evaluating specific behaviour tests due to the direct effect of *T. gondii* infection such as anxiety, object recognition test, open field, social interaction, recognition memory, working memory, and spatial memory [19] have been documented. Interestingly, indirect effect of *T. gondii* in the brain of infected host that will provide us with a snap shot of neurological changes received little attention [14]. This points to a combination of direct and indirect changes to fully understand *T. gondii* infection, suggesting a contribution to mental disorder.

Available reports had implicated *T. gondii* to trigger some neurotransmitters [13,20], with recent and scarce data suggest that the effect may be strain-dependent [21]. The existence of different typical and atypical strains worldwide that varied between geographical location is the major cause of *T. gondii* complication [22,23]. This requires several aspects on the indirect mechanism by which *T. gondii* cysts provokes changes on the dopaminergic cells to be further investigated. Noteworthy, previous studies in Malaysia have reported direct effects of *T. gondii* on rats behaviour that can cause mental disorders such as schizophrenia [24]. However the indirect effect of *T. gondii* tissue cyst on neurotransmitter changes that lead to behaviour changes have not been evaluated. This study was designed to investigate the role of dopaminergic system in the brain of *T. gondii* infected rat's leading to neurotransmitter changes.

## 2 Materials and methods

### 2.1 Animals and experimental groups

Forty-five healthy male Wistar albino rats four weeks old were obtained from a local supplier, Bistari Enterprise, Taman Sri Serdang, Seri Kembangan, Selangor, Malaysia. These rats were randomly divided into five groups containing nine rats each. The experimental groups comprised; experimental group 1, control group, inoculated with phosphate buffered saline (PBS). The experimental group 2, administered with (+) MK-801 hydrogen maleate powder (Sigma-Aldrich, USA), a cognitive impaired model. The experimental group 3, infected with type I strain; experimental group 4, infected with type II strain; experimental group 5, infected with type III strain. The rats were housed three rats per cage and placed under the same laboratory condition and kept at the Animal experimental unit of the Faculty of Medicine and Health Sciences, Universiti Putra Malaysia (UPM). The rooms were ventilated with a temperature of between ±22 °C, and 12 h light-dark cycle for a period of one week to acclimatize. The rats were fed with standard commercially available chow (Gold Coin Feed Mills, Malaysia) with water provided *ad libitum*. All the experimental protocols were conducted according to the Animal ethics guideline and approved by the UPM Animal Care and Use Committee (Supplementary Fig. 1, UPM/IACUC/AUP-R071/2017).

### 2.2 Preparation of *T. gondii* oocyst and infection of rats

The 17 *T. gondii* positive oocysts were obtained from the pet and free roaming cat faeces in Klang Valley, Malaysia [25]. The oocysts were previously identified [24] as positive *T. gondii* strain of type I, II and III. Each oocyst were mixed with 2.5% potassium dichromate - K_2_Cr_2_O_7_ (Sigma-Aldrich, USA) and aerated at room temperature for one week to sporulated [26]. The suspension was washed five times with distilled water and sporulated oocysts were quantified in a Neubauer chamber. The final volume of each suspension was diluted with PBS and adjusted containing 1000 oocysts/ml as previously reported [27]. The experimental group 1 served as a negative control, received PBS; experimental group 2, received MK-801injection; experimental group 3, 4 and 5 received sporulated suspension of type I, II and III respectively. Approximately 0.2 ml per rat of each suspension of type I, II, III and PBS (0.85%) was inoculated once to the experimental groups, 1, 3, 4 and 5 using gavage probe respectively. While experimental group 2 which served as positive control received the drug MK-801(Hydrogen maleate powder, Sigma-Aldrich, USA). The drug was dissolved in PBS and injected subcutaneously with each rat receiving 0.6 mg/kg. This dosage of MK-801 as previously reported can induce/mimic symptoms of mental disorder in rat [27]. The drug was administered twice a day for seven days and behavioural deficits tested 36 h after the last injection [27]. Body weight were measured weekly throughout the entire period of the study.

### 2.3 Behavioural test of rat at nine weeks post infection

The behavioural test which includes fatal feline attraction test and elevated plus maze were carried out at nine weeks post-infection. This period was found in *T. gondii* to established chronic infection in the rat [28,29].

### 2.4 Fatal feline attraction test

The fatal feline attraction test using four-choice odour response test (FCORT) is a measure of innate aversion of the rat to cat urine [30]. The open field apparatus is a squared box made up of Plexiglas that had closed base an open top (75 cm × 75 cm base, 40 cm height). The base of the box was equally divided with parallel vertical and horizontal lines into 25 smaller square units measuring 15 cm × 15 cm with modification. The four corners of the square box contained the odour of different animals soaked in rat's straw bedding using Petri dish. The four distinct odours are the cat (feline) odour, fresh straw bedding containing 15 drops of cat urine; rat (own) odour, own straw bedding; neutral (water) odour, fresh straw bedding containing 15 drops of sterile water and dog (pet) odour, fresh straw bedding containing 15 drops of dog urine [3]. The dog odour as a predatory mammal was used in the present study. This choice is particularly important because the dog is a rat predator similar to cat natural predator [31]. The position of the odour was interchanged between each test to avoid bias [2,3,32]. The behaviour of rats displayed was captured for a duration of 10 min. Two 100 W halogen lamps indirectly illuminated the room. The Logitech camera C350 video camera (Logitech, Malaysia) was fixed to the ceiling directly facing the maze which is connected to a computer laptop (HP® Windows 7 Laptops, U.S.A.) with ANY-Maze video tracking software (ANY-Maze™, Stoelting Co., Chicago, U.S.A.) for automated recording. At the end of each rat test, 70% ethanol and sterile water were used to clean the square box. The time spent near each odour was recorded.

### 2.5 Elevated plus maze

**Fig. 1:**
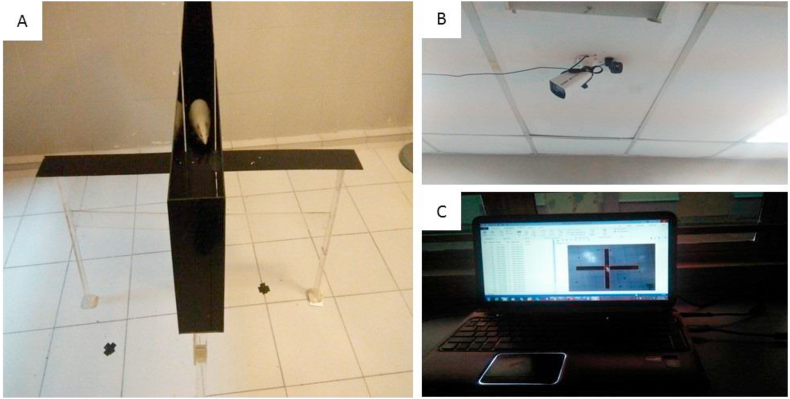
**Image of elevated plus maze test apparatus; A, is a rat in the maze; B, Logitech camera fixed to the ceiling and C, ANY-maze video recording software.** A: This represents the image of a rat inside EPM during behaviour evaluation. B: Logitech camera fixed on the ceiling directly facing the EPM to capture rat movement. C: ANY Maze video tracking software connected with Logitech camera for a recording of rat movement.

The elevated plus-maze (EPM) is a measure of anxiety for rodents (Fig. 1) which consist of a black Plexiglas (A) with two arms crossed at the centre (10 cm × 10 cm) each having equal dimensions. The four arms consist of two open arms (50 cm length x 10 cm width) and two closed arms (50 cm length x 10 cm width x 40 cm height). The height of the maze was elevated 50 cm above the floor [33,34]. Lighting (B) and tracking software (C) was done as described in fatal feline attraction test. The rats were moved into the behaviour room and allowed for 10 min to acclimatize before the test. Each rat was gently removed from the cage and placed at the centre of the EPM facing the open arm and their behaviour was captured for 5 min. After the behaviour test, rats were returned to their home cage. At an interval between each rat test, the EPM was cleaned with 70% ethanol and wiped with sterile water, air dried to remove odour. The time spent, and the number of entries into the open and closed arm were recorded [34,35].

### 2.6 Preparation of rat brain tissue samples

For relative gene expression analysis, a small portion of the brain from the amygdala section was cut using sterile blade into smaller size approximately 1 mm and placed in a 2 ml container that has 1 ml of RNAlater, Thermo Fisher Scientific (Carlsbad, CA, USA) as a preservative. The 2 ml tube containing the brain tissue was kept in an ice cold box which was later stored at −80 °C before subsequent processing.

### 2.7 RNA extraction

Total RNA was extracted from rat brain tissues using RNeasy mini kit (Qiagen Hilden, Germany) according to the manufacturer's instruction. Rat brain tissue was distrupted by a tissue homogenizer Kinematica AG Polytron PT-MR 2100 Benchtop (Hamburg, Germany) all centrifugation was carried using a Benchtop Eppendorf MiniSpin (Hamburg, Germany). Finally, elution was performed twice with eluting RNA from the first elution. All extracted RNA were stored at −30 °C until later use.

### 2.8 Analysing nucleic acid quantity and quality

The RNA concentration was measured in Nanodrop spectrophotometer (Carlsbad, CA, USA). This was followed by denaturing agarose gel electrophoresis to check RNA integrity.

### 2.9 cDNA template preparation (reverse transcription)

The total RNA was normalized to have 100 ng concentration for each sample and reverse transcribed to cDNA using qPCRBIO (PCR Biosystem Ltd; London, UK) kit following the manufacturer's instruction. The converted cDNA was stored in −20 ° C until later use.

### 2.10 Primer optimization on conventional PCR

The primer optimization was performed with a final volume of 25 μl as reaction mixture which contained primer sequences as listed in Table 1. The conventional PCR reaction mixtures contain 12.5 μl of Superhot Master Mix (BIORON GmbH, Germany), 0.2 μM (final concentration) each of forward and reverse primer, 4 μl of cDNA template and PCR grade water. Amplification was performed in a Bio-Rad Mycycler (Thermal Cycler PCR, USA) with an initial denaturation at 95^°^c for 3 min. This was followed by 30 cycles of denaturation at 94 °C for 30 s, annealing for each primer was optimized through gradient protocol for 45 s and extension at 72 °C for 1 min. The final extension was carried out at 72 °C for 10 min. The PCR product and 100 bp ladder (BIORON, GmbH, Germany) was resolved in 1.5% agarose gel stained with ethidium bromide and visualized with Bio-Rad gel doc XR (Molecular imager, USA).

**Table 1:** List of primers for gene expression study in qPCR.

Accession no.	Gene		Primer	Length	Tm	Amplicon size
NM_008324.2	IDO1	F	CCAGTCCGTGAGTTTGTCAT	20	58.57	150 bp
		R	ATCAGTGGGCTTCTTCTTCG	20	59.43	
NM_145949.2	IDO2	F	TACCTCCCTCGTCCCTTAGT	20	57.73	145 bp
		R	CCTGGTGCTTCACAGACA	18	56.57	
NM_010076.3	DRD1	F	GCCAGACCACCACAGGTAA	19	59.54	146 bp
		R	GAAGAAAGGGAGCCAGCA	18	59.04	
NM_010077.3	DRD2	F	CTCCTCCATCGTCTCGTTCT	20	59.40	141 bp
		R	GGTGTCTTCAGGTTGGCTCT	20	59.30	
AY618569.1	Β-Actin	F	TGGCTCTGTGGCTTCTACTG	20	59.30	192 bp
		R	TACCTTCCCAACTCCTCACC	20	59.30	
AB017801	GAPDH	F	ACTCTACCCACGGCAAGTTC	20	59.40	133 bp
		R	TACTCAGCACCAGCATCACC	20	59.30	

### 2.11 qPCR assay development

The qPCR reaction mixture was performed in a final volume of 20 μl that contained primer sequences for the gene of interest (GOI). The same 20 μl reaction mixture was also prepared for each of the two reference genes (RG), GAPDH and B-Actin. The primer sequence for the GOI and the RG are listed in Table 1. The two RG were normalized using the average of the two genes to assess the reproducibility of the assay and the pattern of expression of the GOI. The primers were designed using Primer3 software. The qPCR reaction components contain 10 μl Luna universal qPCR master mix, 0.5 μM (final concentration) each of forward and reverse primer, 2 μl of cDNA template and PCR grade water. For each reaction mixture, a negative control was prepared using a complete reaction mixture without cDNA template. Each sample from the experimental group was prepared in triplicate and carefully dispensed into 96 well plates, sealed with transparent film.

### 2.12 Cycling protocol

Relative gene expression amplification was performed in Eppendorf Mastercycler *ep* realplex *4s* with an initial denaturation at 95 °C for 60 s. This was followed by 40 cycles of denaturation at 95 °C for 15 s, extension at 60 °C for 30 s. Melt curve analysis was also carried out at 60 °C to determine primer specificity.

### 2.13 Gene expression data analysis

The cycle threshold (CT) values obtained were used to calculate the fold change using Livak method [36]. The average (AVG), CT values for each gene of interest (GOI) were normalized with the two reference gene (RG) average CT values [ΔCT = AVG CT GOI – AVG CT RG]. Thus, ΔΔCT (ΔCT treatment – ΔCT control). The fold change of each gene was expressed as 2^^−ΔΔCT^ among the various experimental groups of the rat.

### 2.14 Statistical analysis

GraphPad Prism (version 7.0) software (GraphPad Software, San Diego, CA, USA) was used to analyzed anxiety through one way analysis of variance (ANOVA) followed by Bonferronis’ post hoc test. Data generated from fatal feline attraction by two way ANOVA followed by Bonferroni post hoc test. A comparison was made between the experimental groups with a value of P < 0.05.

## 3 Results

The body-weight of the MK-801 induced model group of mental disorder, *T. gondii* infected groups and control group of the rat were comparable at the end of 8 weeks post infection before the commencement of behavioural test.

### 3.1 Fatal feline attraction test (FFAT)

**Fig. 2:**
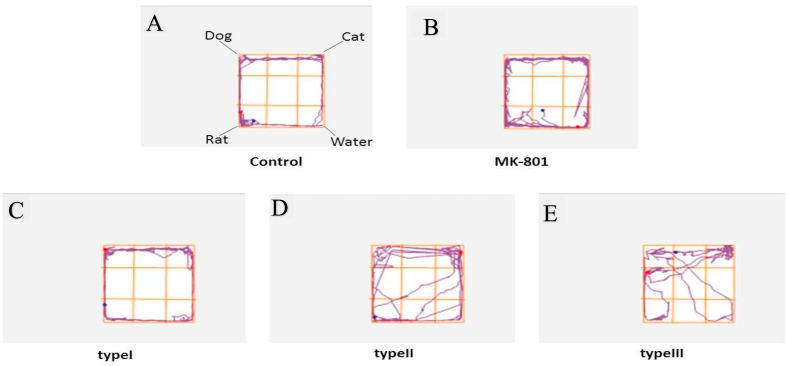
**Representative images of track plots showing the location of cat urine, dog urine, rat urine and water.** Control represents the group of rats ingested with normal saline. MK-801 represents the group of rats induced with a drug as a model of cognitive impairment, while type I, type II and type III represents the *T. gondii* infected groups of rats fed with single genotype strain (n = 6).

**Fig. 3:**
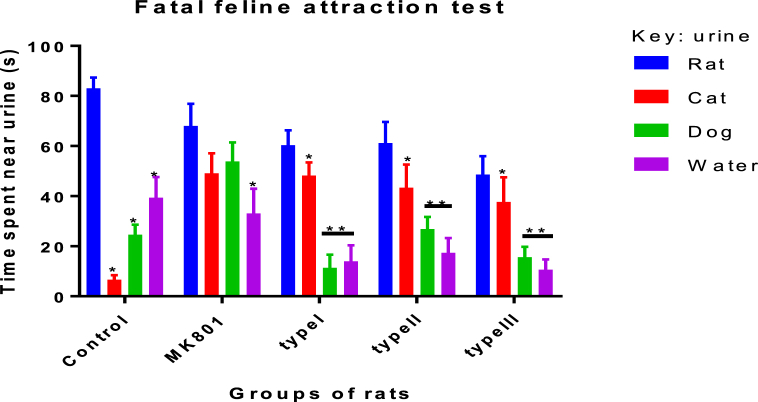
**Effects of Mk-801 induced and *T. gondii* infected groups of rat on attraction or repulsion to different animal's urine.** The time rat spent near each urine is measured as an attraction relative to their own urine. Control, n = 6; MK-801, n = 6 and *T. gondii* infected (type I, n = 6; type II, n = 6 & type III, n = 6). *T. gondii* infected rats spent more time near cat urine compared with control groups of rat, P < 0.05. Error bars indicate SEM.

The attraction of rat to cat scented areas is a suicide mission which facilitates predation by their natural enemy, cat [30]. The result of the present study found that all the three strains of *T. gondii* type I, II and III had a comparable attraction to cat odour in infected rats at nine weeks post infection compared to PBS control group. Representatives of track plots are shown in Fig. 2. The attraction to rat, cat, dog urine and neutral water was significantly different using one-way analysis of variance [*F*_*(3, 16)*_ *= 7.77, P = 0.002*]. In addition, Bonferronis’ post hoc test revealed apart from their own rat odour 64.25 ± 12.61, *T. gondii* infected rats spent a significant number of time near cat urine odour (37 ± 17.57), followed by dog urine (26.47 ± 16.59) and less attraction towards neutral water (22.87 ± 12.61). In contrast, MK-801 induced group of the rat were attracted to all the three odours (Rat, Cat & Dog) with no significant difference (Fig. 3).

### 3.2 Elevated plus maze (EPM)

**Fig. 4:**
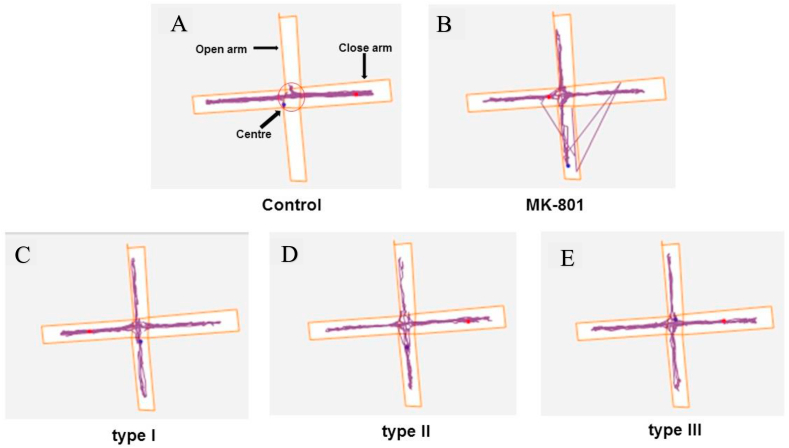
**Representative images of track plots of anxiolytic like behaviour of control, MK-801 and *T. gondii* infected experimental groups of rats in EPM.** Control represents the group of rats ingested with normal saline. MK-801 represents the group of rats induced with a drug as a model of cognitive impairment, while type I, type II and type III represents the *T. gondii* infected groups of rats fed with single genotype strain (n = 6).

**Fig. 5:**
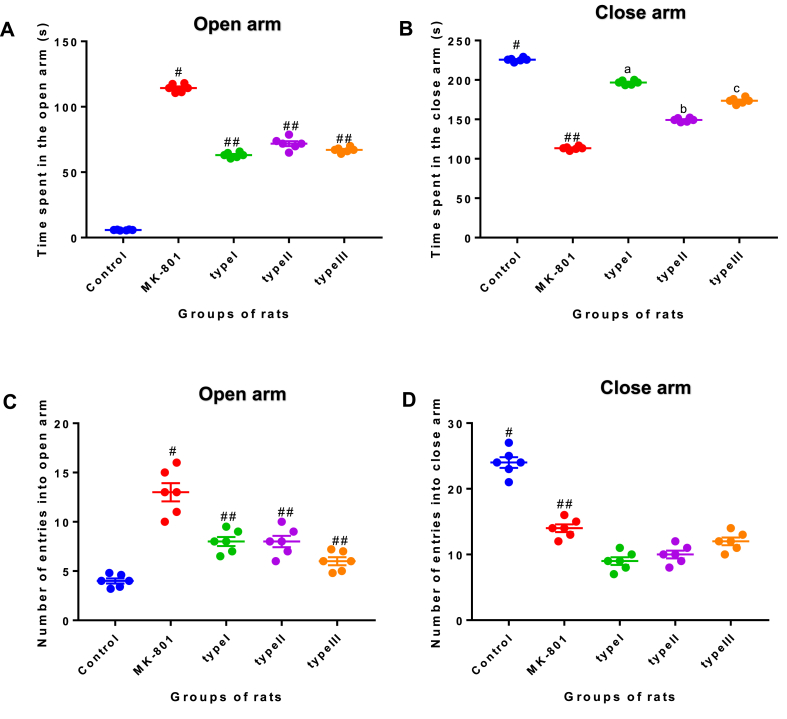
**Effect of MK-801 induced and *T. gondii* infected groups of rats on anxiety-like behaviour in the elevated plus maze.** The experimental groups comprised; experimental group 1, control group, inoculated with PBS. The experimental group 2, administered with (+) MK-801, rat model of cognitive impairment. The experimental group 3, infected with type I strain; experimental group 4, infected with type II strain; experimental group 5, infected with type III strain. (A) Time spent in the open arm as a measure of anxiety. (B) Time spent in the close arm as a measure of anxiogenic. (C–D) Number of entries into open and close arm respectively. Data were analyzed using one-way ANOVA followed by Tukey's post hoc test. Data are presented as a mean ± SEM for 6 rats in each experimental groups (P < 0.05 and 0.01) compared to the control group. ^#^P < 0.05 vs control group, ^##^P < 0.05 vs *T. gondii* infected groups (n = 6).

On the nine week post infection (Fig. 4), Mk-801 induced groups of rats had a significance increase of open arms entries compared to that of controls using one-way analysis of variance [time in open arm *F*_*(4, 25)*_ *= 1182, P < 0.001*; open arm entries, *F*_*(4, 25)*_ *= 34.02, P < 0.001*]. This pattern of open arm entries was also observed to reduce close arm exploration. The representatives of the time spent in an open arm (A), close arm (B) versus entries into open arm (C) and close arm (D) arm in track plot is shown in Fig. 5. In addition, *T. gondii* infected rats showed increased in the exploration of the open arm compared to controls. Further, Bonferronis’ post hoc test revealed *T. gondii* infected with single genotype strain of type I, type II and type III were different to control group in time spent/entries in the open arms (Fig. 5). Thus, the anxiety-like behaviour recorded in the elevated plus maze was not *T. gondii* infected genotype strain dependent.

### 3.3 Effects of *T. gondii* strain of type I, II and III on neurotransmitters

**Fig. 6:**
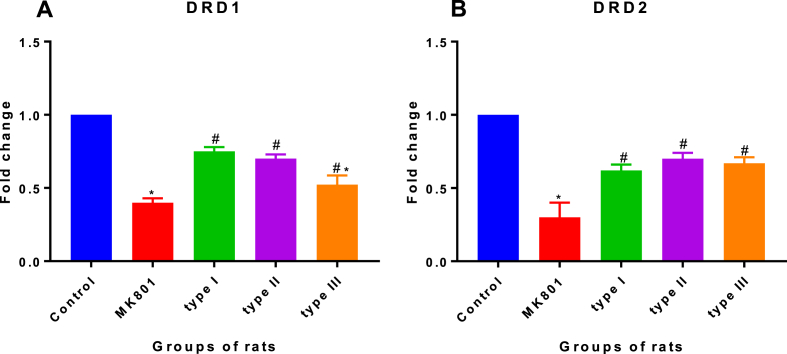
**Effects of *T. gondii* genotype strains on mRNA level of (A) DRD1 and (B) DRD2 genes in the brain of rats.** The experimental groups comprised; experimental group 1, control group, inoculated with PBS. The experimental group 2, administered with MK-801, a rat model of cognitive impairment. The experimental group 3, infected with type I strain; experimental group 4, infected with type II strain; experimental group 5, infected with type III strain. *T. gondii* genotype of type I, type II and type III decreases DRD1 and DRD2 mRNA level (A&B). All data were expressed as mean ± SEM (n = 3). *MK801 vs control, ^#^type I, II and III vs control and ^#@^type I vs type II, type I vs type III or type II vs type III at P < 0.05. Data were presented as SEM.

In this study, mRNA level of the two classical DRD which include DRD1 and DRD2 were evaluated using qPCR. Data obtained were analyzed with One way ANOVA which indicated significant statistical differences in the DRD1 [*F (4, 10) = 46.71, P = 0.001*] mRNA level in the brain of experimental groups of rats. This was followed by Bonferroni's post hoc test which showed a fold change decrease of DRD1 gene transcription in the MK-801 (*0.4 ± 0.05, P < 0.001*) experimental group of rat compared to the normal control (*1.0 ± 0, P < 0.001*) group of the rat. In addition, DRD1 gene transcription showed fold change decrease in type I (0.75 ± 0.7), type II (0.7 ± 0.05) and type III (0.5 ± 0.11) mRNA level n the three *T. gondii* infected experimental groups of rats when compared with control (*1 ± 0, P < 0.001*) experimental group of rat (Fig. 6A). Further, a fold change decrease was observed between MK 801 (*0.4 ± 0.05, P < 0.001*) experimental group of rat mRNA level compared to type I (0.75 ± 0.7) and type II (0.7 ± 0.05) experimental group of rats. However, despite decrease in mRNA level no significant statistical fold change difference was observed between MK801 (0.4 ± 0.05, P < 0.001) and type III (0.5 ± 0.11), and also between type I (0.75 ± 0.7) and type II (0.7 ± 0.05) mRNA level.

The gene transcription study of DRD2 gene (Fig. 6B) followed a similar pattern with that of DRD1 gene transcription. No significant fold change difference was observed between *T. gondii* infected, type I (0.62 ± 0.04), type II (0.7 ± 0.04) and type III (0.67 ± 0.04) groups of rats.

**Fig. 7:**
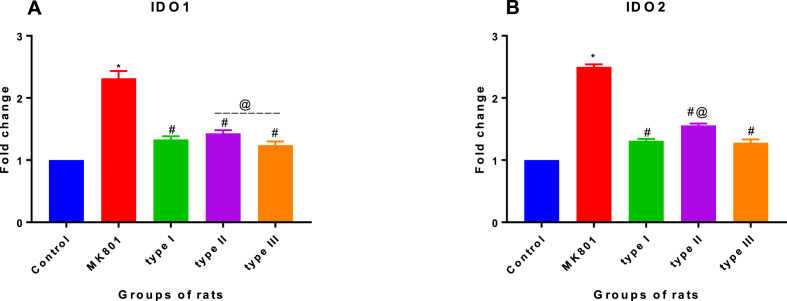
**Effects of *T. gondii* genotype strains on mRNA level of (A) IDO1 and (B) IDO2 genes in the brain of rats.** The experimental groups comprised; experimental group 1, control group, inoculated with PBS. The experimental group 2, administered with MK-801, a rat model of cognitive impairment. The experimental group 3, infected with type I strain; experimental group 4, infected with type II strain; experimental group 5, infected with type III strain. *T. gondii* genotype of type I, type II and type III decreases IDO1 and IDO2 mRNA level (A&B). All data were expressed as mean ± SEM (n = 3). *MK801 vs control, ^#^type I, II and III vs control and ^#@^type I vs type II, type I vs type III or type II vs type III at P < 0.05. Data were presented as SEM.

In the present study, IDO1 fold change was evaluated in qPCR. One way ANOVA revealed differences statistically in fold change of IDO1 [*F (4, 10) = 172.5, P = 0.001*] in the brain of experimental groups of rats. The Bonferroni's post hoc test revealed a fold change increase in IDO1 gene transcription in MK-801 (2.32 ± 0.12, P < 0.001) experimental group of rat compared with the control (1.0 ± 0) of a group of the rat. In addition, fold change increase was observed in type I (1.33 ± 0.049), type II (1.43 ± 0.05) and type III (1.24 ± 0.06) *T. gondii* infected experimental groups of rats compared to the normal control (1.0 ± 0) experimental group of the rat. Alternatively, no significant fold change difference was observed between type I (1.33 ± 0.049) and type II (1.43 ± 0.05) and also between type I (1.33 ± 0.049) and type III (1.24 ± 0.06). This is shown in Fig. 7A.

The IDO2 gene transcription (Fig. 7B) when analyzed also revealed a similar pattern to that of IDO1 fold change. No statistical significant fold change difference was observed between the type I (1.31 ± 0.03) compared with type III (1.28 ± 0.053) experimental groups of the rat.

### 3.4 Discussion

In the present study, while *T. gondii* infected groups of the rat with single type I, II and III strains show similar attraction to cat urine odour only. MK-801 induced group of rat spent comparable time near cat and dog urine scented areas, while PBS control group avoided all scented areas and spent considerable time near own urine odour. These results obtained from this study are consistent with previous findings in *T. gondii* infected rodents that reported attraction to cat odour [28,37,38]. Further, study by Kannan et al. [31] have found *T. gondii* infected with PRU strain attracted to cat urine but not ME49 at 7 month post infection. However, this present study observed comparable attraction to cat odour by all the three *T. gondii* strain of type I, II and III. The use of four different odours in the previous study were largely using cat (predator), rat (own), rabbit (non-predator) and neutral water [28,37,38], however the present study employed dog (a pet) urine instead of rabbit urine. The use of dog urine is in line with a study that tested attraction of *T. gondii* strain of PRU and ME49 infected rat on a two-choice (dog & cat odour), [1]. Their findings of cat over dog odour attraction were similar to what we found in this study, except that MK-801 induced group of rat showed comparable attraction to both odours. The data in this study also pointed out that *T. gondii* infected rat attraction to cat urine is highly specific and facilitate transmission compared to the result of MK-801 induced group of rat. Nevertheless, different *T. gondii* strain, rodent species and time allotted for the odour test was not uniform among studies and likely remained future challenge, but the present study had moved closer to explain the aetiology of mental illness.

Further, MK-801 (Model) induced and *T. gondii* infected group of rats were observed to have reduced level of anxiety that caused them to explore more open arms of elevated plus maze compared to PBS control group. This is consistent with previous findings in the rat [33] which reported reduced levels of anxiety, that makes them more prone to predation by their natural enemy, felids [39]. In addition, studies have also documented anxiety-like behaviour in mice infected with different genotype strain of *T. gondii* [1,40]. Nevertheless, the present study found comparable anxiolytic effect of *T. gondii* infected groups of rats with (Fig. 5) type I, II and III strain. The previous studies have also reported similar behavioural changes caused by *T. gondii* type II strain of PRU and ME49 infected mice [1]. However, Bezzera et al. [21] demonstrated that mice infected with *T. gondii* VEG (type III) strain showed more behavioural changes compared to ME49 (type II) strain. On the other hand, Vyas et al. [29] reported that male rat infected with *T. gondii* did not alter anxiety level that can lead to behaviour changes. The discrepancy between studies as related to the anxiety-like behaviour caused by *T. gondii* infection may likely relate to parasite infective stage and the age of the laboratory animals during behaviour test. Gonzalez et al. [33] found behaviour changes in rats infected with RH strain of *T. gondii* at three weeks compared to seven weeks post infection, which is less than nine weeks of the present study. However, the three *T. gondii* genotype strains of type I, II and III used in this study were not able to show different behaviour changes in rats. *T. gondii* genotype strains varied among geographical location that requires special attention because of their virulent properties [41], stage causing the infection [42] and immune status of the host [43].

In this present study, we also report *T. gondii* infection in male Wistar rats and MK-801 induced neurotransmitter of dopamine receptors (DRD) changes in the brain, which implies dopamine (DA) concentration. The present results showed decrease in gene transcription of DRD1 and DRD2 in M-K801 and *T. gondii* infected rats compared to PBS control, suggesting DA is likely hyperactive in the mesolimbic and hypoactive in the mesocortical pathway (Fig. 6A and B). This is consistent with the findings of Wang et al. [44] who observed a decrease in DRD gene transcription in *T. gondii* infected mice brain compared with the normal control group inoculated with PBS. Although DA was not measured in this study, but the previous study have demonstrated that when DRD gene transcription was decreased, for example in *T. gondii* infected mice, (DA) concentration rises due to insufficient DRD binding sites [44]. Interestingly, a study in rats also showed increased DA in *T. gondii*-infected and amphetamine administered rats [45]. Further, a study in mice infected with *T. gondii* have also reported an increase in DA synthesis in brain regions [7]. In contrast, studies of dopaminergic cells *in vitro* showed a decrease in DA as a result of stress or destruction of the host cell [12]. In line with the DA hypothesis, DRD antagonist like MK-801 is a classical activator of DA release which can cause certain psychotic symptoms in healthy individuals that trigger symptoms of schizophrenia [46]. Nevertheless, the present result further show high similarity of DRD1 gene transcription in type III *T. gondii* infected group of rats compared to MK-801 model group of rat, than rat infected with either type I or II strain. This is in agreement with the previous finding which reported that type III strain is likely to cause behaviour changes compared to type II [21]. However, the result of DRD2 gene transcription revealed a significant difference between MK-801 model groups of rat compared with *T. gondii* infected group of rats (type I, II and III) indicating a comparable effect on behaviour changes in *T. gondii* infected groups of rats. Thus, *T. gondii* infection in both rats and mice on neurotransmitter changes have varied extensively among studies. It is necessary to note the effects of different experimental designs which ultimately produce variable outcomes. The present study is the first to report DRD1 and DRD2 genes deficits in *T. gondii* infected rats.

Further, the present study showed elevated levels of IDO1 and IDO2 in the brain of *T. gondii* infected rats, suggesting that KYNA was upregulated (Fig. 7A and B). Consistent with the previous studies in mice, *T. gondii* infection increase in IDO level [[46], [47], [48]]. Interestingly, KYNA was found significantly increased in the brains of humans with symptoms of schizophrenia [47,49], reflecting the contribution of the compound to cognitive impairment. In a recent study in mice, Wang et al. [44] found an increase in IDO two-fold among the *T. gondii* infected mice compared with the normal control group of mice inoculated with PBS [44]. There is a consensus that IDO genes dysregulation as part of neurotransmitter activity play a role in the pathogenesis of the psychotic disease, but the pathway involved was not clear [10,16,50]. Moreover, an imbalance of IDO1 and IDO2 gene transcription in the brain system has been linked to tryptophan degradation which can alter the serotonergic neurotransmission across certain compounds like kynurenic acid [10,50]. Therefore, the result of the present study indicates that *T. gondii* infection may likely cause a brain dysfunction leading to behaviour changes similar to those previously reported [24] when *T. gondii* infected rats were subjected to behavioural test related to schizophrenia-like conditions.

Lastly, the data in this study revealed a decrease in DRD1 and DRD2 genes transcription as a binding site for DA transmission in the brain of *T. gondii* infected rats, which means that the lower the DRD1 and DRD2 gene transcription, the DA concentration may likely increase in the mesolimbic and decrease in the mesocortical pathway. In contrast, elevated levels of IDO1 and IDO2 in the brain of *T. gondii* infected rats can possibly regulate the levels of neurotransmitters associated with KYNA biosynthesis. Here we examined the relative expression of DRD1, DRD2 and IDO1 and IDO2 genes transcription in MK-801 model schizophrenia group and *T. gondii* infected rat brain and subsequent neurotransmitter changes. The important limitation in this study is that it evaluated body weight changes, fatal feline attraction, fear and anxiety and neurotransmitter levels only. However, object recognition test, social interaction test, locomotor activity, learning and memory analysis remains to be determined.

## 4 Conclusion

In conclusion, *T. gondii* infected rats shows behaviour changes at nine weeks post infection. The *T. gondii* infection changes the neurotransmitter level through dysregulation of DRD1, DRD2 and IDO1, IDO2 in the brain. In the MK-801 cognitive impairment model group of rat, the DRD is decreased and IDO is elevated in the brain which is closely related to the three *T. gondii* infected groups compared to PBS control group. The decrease in DRD gene transcription in *T. gondii* infected rats close to MK-801 model group may likely suggest that DA activity is increased in the mesolimbic and decreased in mesocortical pathway resulting into behaviour changes mimicking schizophrenia-like condition. This results further strengthen the assumption of cognitive impairment in *T. gondii* infected rats. Although more specific behavioural tests such as object recognition test, social interaction test, locomotor activity, learning and memory analysis were outside the scope of this study, it is important and should be considered in future research. Thus, these findings may have provided not only the effects of *T. gondii* infection on neurotransmitter but also the mechanisms underlying the indirect effect of *T. gondii* tissue cysts leading to cognitive impairment.

## Ethical issues

All the experimental protocols were conducted according to the Animal ethics guideline and approved by the UPM Animal Care and Use Committee with reference code: UPM/IACUC/AUP-R071/2017.

## Declarations

### Author contribution statement

Mohammed Nasiru Wana: Conceived and designed the experiments. Malaika Watanabe, Shariza Nordin, Rusliza Basir: Conceived and designed the experiments; Performed the experiments; Analyzed and interpreted the data; Contributed reagents, materials, analysis tools or data. Samaila Musa Chiroma, Sharif Alhassan Abdullahi: Conceived and designed the experiments; Performed the experiments; Analyzed and interpreted the data; Contributed reagents, materials, analysis tools or data; Wrote the paper. Ngah Zasmy Unyah, Mohamad Aris Mohd Moklas, Roslaini Abd. Majid: Conceived and designed the experiments; Analyzed and interpreted the data; Wrote the paper.

### Funding statement

This work was supported by the 10.13039/501100003093Ministry of Higher Education, Malaysia by supporting this work through the Fundamental Research Grant Scheme (FRGS): 5524777.

### Data availability statement

Data will be made available on request.

### Declaration of interest's statement

The authors declare no conflict of interest.
