# Supplementary figures and images for: Toxoplasma gondii induced cognitive impairment in rats via dysregulation of dopamine receptors and indoleamine 2,3 dioxygenase
Source: Heliyon. 2023 Mar 9;9(3):e14370. doi: 10.1016/j.heliyon.2023.e14370 (PMC10025920; doi:10.1016/j.heliyon.2023.e14370)

**Supplementary File**

**
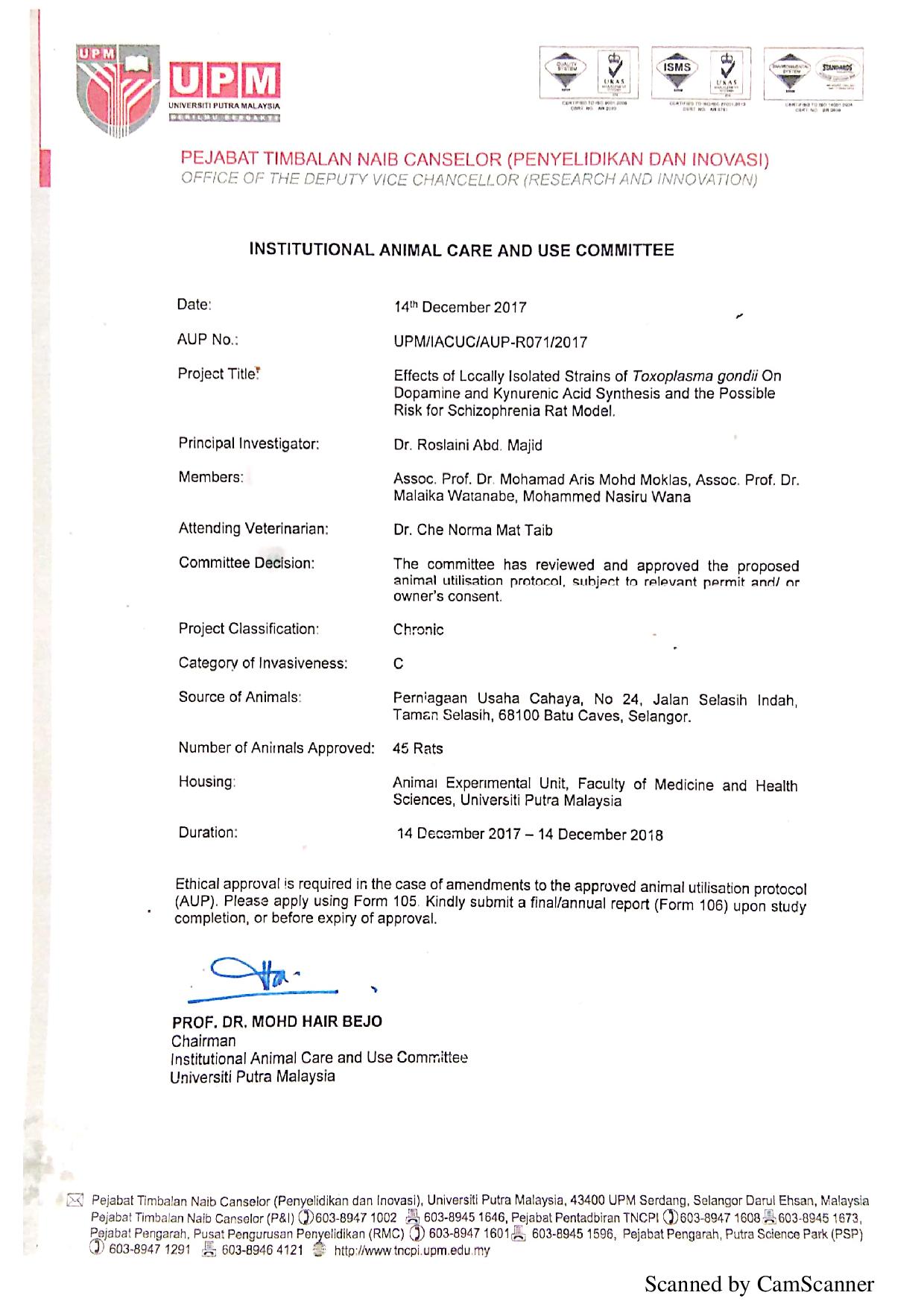
**

**Figure 1: Animal ethics approval**

Supplement: Multimedia component 1 [file mmc1.docx]
